# Anti-Müllerian hormone levels are associated with skeletal maturity in adolescent girls in the Fels Longitudinal Study

**DOI:** 10.21203/rs.3.rs-3342941/v1

**Published:** 2023-09-29

**Authors:** McKenzie L Ford, Audrey C Choh, Brandon Gonzalez, Steven R Lindheim, Frank Z Stanczyk, Lynda K McGinnis, Stefan A Czerwinski, Miryoung Lee

**Affiliations:** University of Texas Health Science Center at Houston; University of Texas Health Science Center at Houston; University of Houston; Wright State University; University of Southern California; University of Southern California; The Ohio State University; University of Texas Health Science Center at Houston

## Abstract

The role of anti-Müllerian hormone (AMH), a potential marker of the hypothalamic-pituitary-ovarian axis, is not well established in adolescent females. Most studies use secondary sexual characteristics or chronological age as predictors for AMH. Skeletal maturity, an indicator of bone development, has not been examined to predict AMH. This study sought to examine patterns of change in AMH in relation to skeletal maturity. Demographics, anthropometry, hand-wrist radiographs, and cardiometabolic risk factors from 88 females (212 observations), between the ages of 8 to 18 years from the Fels Longitudinal Study were used in this study. AMH was analyzed using ELISA from stored frozen serum samples. Generalized linear mixed effect modeling was used. In the stepwise regression models, log-transformed AMH (AMHlog) was regressed on relative skeletal age as the skeletal maturity indicator (calculated as chronological age minus skeletal age) and adjusted for chronological age, adiposity, and cardiometabolic risk factors. Skeletal maturity significantly predicted lower AMHlog (β= −0.073, SE=0.032, p=0.023). Glucose was significantly associated with decreases in AMHlog (β= −0.008, SE=0.004, p=0.044). Chronological age modeled as a cubic function was not significant. AMH and skeletal maturity may provide correlated information on growth and pubertal status in adolescent females.

## Introduction

The function of the anti-Müllerian hormone (AMH) varies depending on the sex and life stage of an individual. During prenatal development, AMH is considered essential in the sex differentiation of a fetus and the absence of AMH plays a role in developing Müllerian duct into female reproductive organs ^1-3^. To date, the majority of AMH research has been conducted regarding fertility and ovarian reserve in adult women ^4-9^. AMH, which is produced in the granulosa cells that surround the oocyte, is secreted during the preantral and small antral phases of follicular development. During aging, there is a decrease in production and serum levels of AMH ^3^. In adolescent females, AMH research primarily focuses on polycystic ovarian syndrome (PCOS), where findings suggest that AMH levels are elevated in adolescents with PCOS ^10-12^. Pediatric conditions of other menstrual and chromosomal conditions, such as oligomenorrhea and Turner syndrome have also been researched to understand ovarian function better ^13-17^.

Patterns of AMH in the context of growth and development in adolescent females have yet to be studied as well. We previously reported AMH to have a significant, non-linear relationship with chronological age in relatively healthy female children between the ages of 8 and 18 ^18^. Similarly, Fong *et al.* found AMH levels increase from infancy until adolescence, reach a peak at 15.8 years of age, plateau until 25 years of age, and then begin to decline gradually ^19^. These results suggest that childhood follicular dynamics may differ from that of adulthood. Furthermore, notable inter-individual differences in AMH levels were reported across all chronological ages ^19^. In contrast, Hagen *et al.* showed that intra-individual AMH levels only undergo minor fluctuations throughout childhood and adolescents. Therefore, the authors suggest that a random AMH measurement would likely be representative for adolescent females and that AMH levels may indicate ovarian reserve in female children, similar to the relationship identified in adult females ^20^.

The role of AMH in growth and development is further complicated by other risk factors associated with reproductive health and adiposity. Ortega *et al.* reported that females who had early menarche have higher levels of AMH than those found in adult women with regular ovulatory cycles ^21^. However, girls with anovulatory cycles, who tended to have higher AMH levels, were responsible for this trend. It remains unclear if AMH is the cause or consequence of anovulatory cycles ^21^. Adiposity can also affect the non-linear relationship of AMH with chronological age. During the pubertal transition, children with higher waist-to-height ratios had elevated AMH levels even after adjusting for chronological age ^18^.

These inconsistencies in the literature suggest that a better understanding of the role of AMH at different developmental stages, as opposed to chronological age, is still needed. One method to measure adolescent maturity is skeletal age, which assesses the developmental processes of bone ^22^. As such, skeletal age is a tool that can be utilized as an alternative method for evaluating growth and maturity in children. Skeletal age determination is a relatively non-invasive method that typically relies on radiograph images ^23^ and is reported in years, similar to chronological age. Skeletal age is a relatively precise and reliable method for evaluating maturation ^22,24^.

While AMH levels have been monitored across chronological ages, their levels have yet to be examined in association with skeletal age. The aim of this study was to 1) examine the pattern of change in serial measurements of AMH by bone age and in chronological age and 2) to assess the influence of maturation on AMH levels while adjusting for factors associated with maturation in adolescent girls using serial data from the Fels Longitudinal Study. A better understanding of childhood AMH levels may prove useful in explaining or predicting AMH levels later in adulthood and, therefore, providing more insight on female fertility and the ovarian reserve.

## Results

### Participants’ characteristics at the first study visit

At the first visit, AMH levels ranged from 0.46 to 16.20 ng/mL, had a mean of 4.85 ng/mL, and a median of 3.65 ng/mL. Chronological age at the first visit ranged from 7.71 to 16.91 years with an average of 11.61 years and a standard deviation (SD) of 2.75 years. Bone age at first visit ranged from 7.05 to 18.00 years with an average of 12.19 years and a SD of 3.10 years. The average relative skeletal age at first visit was −0.57 years with a SD of 1.19 years and ranged from −3.37 to 3.01 years. Approximately 16% of participants were in early maturing group at the first visit, 69% of participants were in normal or average maturing group, and 15% of participants were in late maturing group. The majority of participants (72.7%) were of normal or healthy weight, 3.4% were underweight, 11.4% were overweight, and 12.5% were obese. Additional descriptive characteristics at baseline are shown in Table 1.

**Figure 1:**
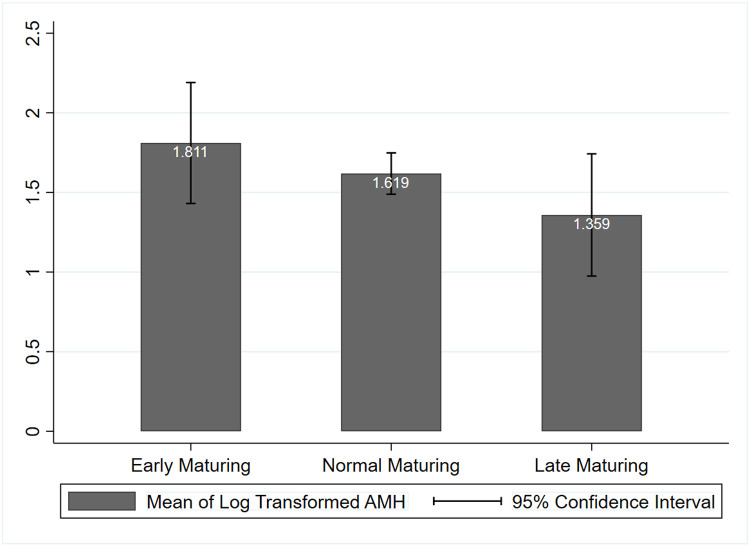
Mean Log Transformed AMH by Relative Age Categories at the participants’ first visit. One way ANOVA omnibus test indicates that the groups are not statistically different from each other.

Mean AMH_log_ values were graphed by relative skeletal age maturation groups at first visit. Figure 1 depicts the cross-sectional inverse relationship between mean AMH_log_ values and the maturation group. Results from a one-way ANOVA, however, revealed no significant differences in mean AMH_log_ values across the three maturation groups at first visit (p = 0.108).

### Longitudinal relationships between AMH levels and age parameters

**Figure 2:**
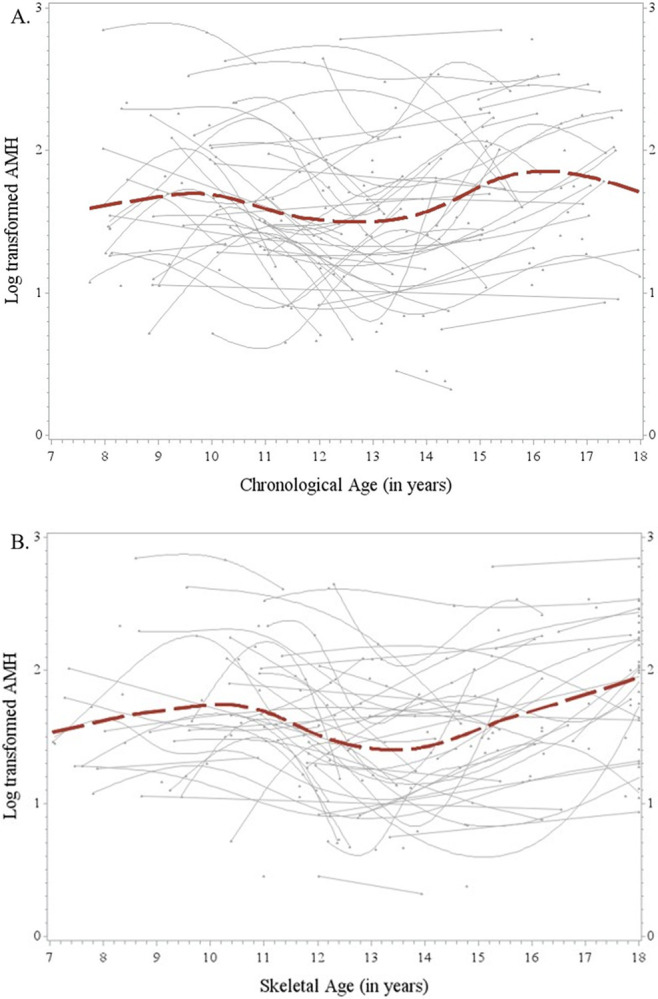
Longitudinal Plot of Log Transformed AMH by Chronological Age (A) and by Skeletal Age (B). Each gray (light) line represents the smoothed longitudinal relationships between log transformed AMH and age (chronological age in 2A and skeletal age in 2B) in each study participant. The dots without connecting gray lines represent the study participants without serial data. The dotted red line represents the smoothed population mean log-transformed AMH levels over the examined age range in the overall sample.

Figure 2 shows the longitudinal relationship between AMH_log_ and chronological age (Figure 2A) and AMH_log_ and with skeletal age (Figure 2B). There appeared to be three inflection points in the AMH_log_ and chronological age relationship. Lower AMH_log_ levels were found around 13 years of chronological age (Figure 2a). The plot of AMH_log_ by skeletal age, as seen in Figure 2b, displayed two points of inflection and also revealed lower AMH_log_ values at around 13 years of skeletal age.

### Generalized linear mixed effect analysis of relative skeletal age and related factors of AMH levels

Table 2 shows the 5 generalized linear mixed effect models. All mixed effect models were significant with an overall omnibus p-value of less than 0.0001. The fixed effects from Model 0, which only consisted of chronological age, chronological age^2^, and chronological age^3^ was not statistically significant, although the random effects were. The −2Loglikelihood (−2LogL) Model 1, which included relative age, chronological age, chronological age^2^, and chronological age^3^ was 256.3. Relative age had a beta coefficient of −0.089 (SE = 0.030) and was significant with a p-value of 0.003.

To build onto Model 1 and account for adiposity characteristics, four adiposity variables were separately included in the second mixed model (Model 2). None of the adiposity variables (BMI percentile (p = 0.186), waist circumference (p = 0.925), waist to height ratio (p = 0.251), and total percent body fat (p = 0.178)) were significant at α=0.05 level. Although none of the adiposity variables considered were significant, total % body fat was added into Model 2 as it had a higher −2LogL value (i.e., lowest p-value) compared to the other adiposity variables at 248.8. Relative age had a beta coefficient of −0.065 (SE = 0.032) and remained significant in the second model with a p-value of 0.042 (Table 2). In Model 3, 15 additional cardiometabolic variables were each considered individually for the final mixed effect model (Model 3). Fasting glucose had a beta coefficient of −0.008 (SE = 0.004) and was significant in the model with a p-value of 0.044 and the model had a −2LogL of 247.3. Thus, glucose was incorporated into the final model (Table 2). None of the other cardiometabolic variables were significant or included in the final model. In this final model, relative age had a beta coefficient of −0.073 (SE = 0.032) and was significant with a p-value of 0.023 (Table 2). Finally, we modeled only bone age, bone age^2^ and bone age^3^ to compare against the Models 0 and 1 to determine which model had the best fit and was the most parsimonious.

The Likelihood ratio tests (LRT) indicate that the relative age parameter is significant in Model 1 and that the addition of relative skeletal age improves significantly and parsimoniously the fit of Model 0 (Model 1). Overall Model 4 was more parsimonious than Model 1. These results suggest that while using bone age along better explains AMH levels. However, it is more difficult to interpret and measure. On the other hand, using chronological age and a measure of skeletal maturity (i.e., relative skeletal age) simultaneously is more easily interpreted while model fit is not as bad as chronological age .

## Discussion

Non-linear patterns were identified for AMH_log_ by skeletal age and chronological age in female children and study results indicated that the relationship between skeletal age and AMH_log_ showed similar patterns to that of the previously reported chronological age and AMH_log_^18^. In our previous study, we found a significant, non-linear relationship between AMH levels and chronological age. These results suggest that declines in AMH levels in children may not reflect a diminished ovarian reserve contradictory to what has been identified in adults.

In our study, peak AMH_log_ occurred near 16 years of chronological age, which was similar to the maximum AMH level value of 15.8 years. Similarly, Fong *et al.* also reported a plateau of AMH level throughout adolescence in their longitudinal plot of AMH by chronological age ^19^. We cannot directly compare our results to Fong *et al.* because the age of our study participants do not extend to 25 years. However, the results from our study identified three likely inflection points in AMH_log_ levels when plotted by chronological age, while plots against skeletal age showed two inflection points. We suspect that the predicted line in the plot of AMH_log_ by chronological age (Figure 2A) may be misleading as there are a few data points closer to the maximum chronological age of 18 years, and may reflect participants who are biologically less mature than their chronological age would indicate. Thus, the downward curve as it approaches this maximum value may be due to a few individuals who happen to have lower values of AMH_log_ due to their potentially lower bone ages. For this reason, it is possible that the true relationship of AMH_log_ by chronological age would more closely mirror the plot of AMH_log_ by skeletal age (Figure 2B), which has only two inflection points. Fong *et al.* also noted considerable inter-individual differences in AMH levels. This observation, combined with the relatively small sample size of this study, could provide some explanation as to why the plot for our study varies from the findings Fong *et al.* ^19^.

The generalized linear mixed effect model (Model 3, Table 2) revealed that for every one-year increase in relative skeletal age, AMH_log_ decreases by 0.073 (1.07 ng/mL in AMH) after controlling for all other variables. Findings also suggest a possible decrease in AMH_log_ levels in individuals who experience late maturation based on the tempo of growth categories at participants’ first study visit. However, our cross-sectional results did not show any statistically significant relationship between AMH_log_ and delayed skeletal maturation. These differences between the cross-sectional and longitudinal analysis may be due to the increased power in the longitudinal study design, and its ability to incorporate interindividual variability in the analysis.

As mentioned previously, AMH research in adolescents predominantly focuses on populations with pre-existing conditions related to sexual maturity, hormones and fertility ^10-12,14,20,25,26^ and, though limited, research on AMH and growth and maturation has typically evaluated sexual characteristics ^27-29^. Research regarding AMH in healthy adolescent populations using other maturational indicators is limited. Our current study is the first to characterize the relationship between the role of AMH and skeletal age throughout childhood of adolescent females. We further explored how AMH levels relate to skeletal maturity, a maturation indicator of the somatic and skeletal growth rather than the maturation of secondary sex characteristics (e.g., breast development).

Regarding cardiometabolic risk factors, after holding all other variables constant, we found a one-unit increase in glucose was associated with a decrease of 0.008 in AMH_log_ (1.01 ng/mL in AMH). That is, AMH_log_ had a significant, negative relationship with glucose. In a study using normal and overweight-obese adolescent females, investigators found positive relationships between AMH and fasting glucose ^30^ while others found no correlation ^31^ or a negative relationship ^32^. In a healthy, general population of adolescent females, cardiometabolic risk factors were not found to be associated with AMH levels ^33^. We also found that there was no significant association between other metabolic factors including high body fat and AMH levels. Due to the inconsistencies regarding the association between cardiometabolic risk and AMH in the literature, particularly among healthy populations, it appears that much is still unknown about the true relationship between AMH and glucose.

A strength of this study included its longitudinal design allowing for the temporal analysis of AMH over several years. Also, along with increased precision and accuracy, the Fels method for the hand-wrist is an appropriate skeletal aging method for this study sample of adolescent females in the United States because the skeletal ages from which the Fels method was created are comparable to that obtained from United States national surveys ^34^. Additionally, the examination of a multitude of adiposity and cardiometabolic variables in growing children allowed us to incorporate these variables into the modeling and to account for biologically relevant relationships that have been identified in the literature in relation to AMH levels. Furthermore, this study may serve as a foundation for further examination of AMH and skeletal maturation as the relationship between skeletal age and AHM levels had not yet been previously described. As this study provided a better understanding of patterns of AMH levels in growing adolescent females, it also has the potential to provide insight for future studies on fertility and ovarian reserve.

The advantages of using skeletal age as an indication of maturation have been noted in many pediatric epidemiologic studies ^35^. However, one limitation of using skeletal age as a growth indicator is that it may only be obtained up until hand ossification is completed. Once the skeletal age of a participant reaches 18 years it cannot exceed that age. A second limitation in this study is that the study sample included only 88 participants, despite the multiple observations available for approximately 60% of the participants. Thirdly, we are unable to generalize the results of this study to a wider, more diverse population as the Fels Longitudinal study consists of largely middle-class, non-Hispanic white participants.

In conclusion, this study is the first to describe the relationship between AMH and skeletal age. Additional research is warranted to corroborate our study findings and characterize the relationship that AMH has with skeletal maturation in adolescent females. Future studies should longitudinally analyze the relationships of AMH with skeletal age and chronological age, as well as the influence of skeletal maturation on AMH level in samples that are representative of the general population of healthy adolescent females.

## Methods

### Study design and participants

This study was a retrospective secondary data analysis that utilized data from the Fels Longitudinal Study. The Fels Longitudinal Study originated in 1929 in Yellow Springs, Ohio and continues to this day. It is the world’s oldest continuous study of growth, development, and aging ^34^. Fels Longitudinal Study participants are followed from enrollment, which is usually birth, until death or infirmity rendering their continued participation impossible ^34^.

The study participants comprised of female children who had serial measurements of AMH and had data for skeletal age. A total of eighty-eight girls who were assessed at least once from 8 years of age to 18 years of age (total observations = 212) were included in the analysis. Due to the serial nature of the data collection method, there was not a standard number of observations per participant. Each participant was observed at least once but no more than six different occasions (range = 1-6 visits, median number of visits = 2). The average duration between the first and last visit was 4.67 years with a minimum of 1 year and a maximum of 9 years.

Participants’ parents/guardians signed a written consent form and adolescents in the study provided verbal assent at each study visit. The data for this study were collected from 1990 through 2014. The Wright State University Institutional Review Board (IRB) approved all study protocols for the Fels Longitudinal Study (Human Subject Protocol 3187). The Committee for the Protection of Human Subjects (CPHS) at the University of Texas Health Science Center in Houston (UTHealth) approved the use of de-identified data and waived the obtainment of informed consent for the present study under HSC-SPH-17-0262.

### Measures

#### Anti-Müllerian hormone assessment

Overnight fasting serum samples were collected from Fels Longitudinal Study participants by means of venipuncture following a minimum eight-hour fast and were stored at −80°C. The Reproductive Endocrine Research Laboratory at the University of Southern California, Los Angeles, California conducted biomarker assays after the frozen, never-thawed samples were shipped from the collection site (Wright State University) in Dayton, Ohio. The Ultrasensitive AMH enzyme-linked immunosorbent assay (ELISA) kit (Ansh Lab, Webster TX) was used to measure AMH levels. More details about this method can be found in Smith et al.^23^ An assay sensitivity of 60 pg/mL was used and the inter-assay coefficient of variation was 9.7% at 1.6 ng/mL and 12.0% at 4.5 ng/mL ^18^.

#### Skeletal age assessment

Skeletal age was collected at each observation using the Fels method for the hand-wrist. Briefly, the Fels method for hand-wrist combines 85 grade maturity indicators and 13 epiphyseal to diaphyseal ratios for bones in the hand and wrist to create a single skeletal age estimate along with a standard error ^36,37^. The chronological age and sex of an individual determines how many maturity indicators are evaluated. Although some indicators are assessed on a maximum five-grade scale, most are either present or absent. The Fels method of skeletal aging is highly replicable with little inter-assessor difference compared to other skeletal age assessment methods ^24^.

Our primary exposure of interest was relative skeletal age, which was created by subtracting a participan’s skeletal age from their chronological age at the same point in time. Thus, positive numbers represent girls whose skeleton are less mature than their chronological age would suggest, suggesting some delayed or late somatic maturation, while negative numbers represent girls whose skeleton are more mature than their chronological age suggests, suggesting earlier somatic maturation ^38,39^. Using relative age enables us to avoid the dependency between bone age (BA) and chronological age. Despite this, we also modelled chronological age by itself and bone age by itself and examined the likelihood ratios for model fit and parsimony and to determine added effect of relative skeletal age compared to chronological age in predicting AMH levels. To illustrate the cross-sectional association between AMH and maturation, we also grouped participants into three categories ^39,40^. If the relative skeletal age of a participant was greater than one standard deviation (SD) above the mean, the participant was considered to be late maturing. If relative skeletal age of a participant was greater than one SD below the mean, females were considered to be early maturing at the first visit ^39^.

#### Covariates

Adiposity variables including body mass index (BMI) percentile, waist circumference, waist to height ratio, and total percent body fat from dual energy X-ray absorptiometry^18^ were measured at each study visit. BMI percentile was categorized using the Centers for Disease Control and Prevention’s guidelines for defining childhood obesity where underweight was less than the 5^th^ percentile, normal or healthy weight was greater or equal to the 5^th^ percentile but less than the 85^th^ percentile, overweight was greater or equal to the 85^th^ percentile but less than the 95^th^ percentile, and obese was greater or equal to the 95^th^ percentile.

Cardiometabolic risk factors have been known to be associated with growth and sexual maturation ^41^. These variables included the homeostatic model assessment of insulin resistance (HOMA-IR) index, glucose, insulin, systolic blood pressure (SBP), diastolic blood pressure (DBP), triglycerides, total cholesterol, high-density lipoprotein cholesterol (HDL-C), and low-density lipoprotein cholesterol (LDL-C),. Detailed information regarding the measurements of cardiometabolic risk factors can be found in Remsberg *et al.*^41^ and Limon *et al.* ^42^

### Analytic Plan

All results were considered significant at a p-value less than 0.05. STATA (version 16) and SAS (version 9.4) were used for all data analyses.

As AMH levels were not normally distributed, the variable was natural log transformed for all analyses. One-way ANOVA was used to compare the three maturing categories according to the relative skeletal age at participants’ first visit, using mean log transformed AMH (AMH_log_) values across the early, average, or late maturing categories. AMH_log_ observations were plotted against both chronological and skeletal age to examine patterns of variability over time by two timing variables.

Generalized linear mixed effect modeling was conducted utilizing a stepwise forward approach consisting of five statistical models. Model 0 included chronological age, chronological age^2^, and chronological age^3^. Model 1 included model 1 parameters plus relative age. Model 2 included all variables from Model 1 with the addition of one adiposity variable from four adiposity variables (BMI percentile, waist circumference, waist to height ratio, or total % body fat). Model 3 included all variables from Model 2 with the addition of significant cardiometabolic risk factor from the pool of cardiometabolic risk factors. A single cardiometabolic variable was included in the model to avoid multicollinearity. Model 4 included bone age, bone age^2^ and bone age^3^. All generalized linear mixed effect models included a random intercept and an unstructured covariance matrix adjusting for unbalanced serial correlated observations. The likelihood ratio test was used to determine model parsimony and whether or not certain covariates were significant. All methods and analyses were performed in accordance with the Wright State University IRB and UTHealth CPHS guidelines and regulations.

## Figures and Tables

**Table 1.** Baseline characteristics of study participants (n=88)

Continuous variables		Mean	SD	Min	Max
Anti-Müllerian Hormone (AMH, ng/mL)		4.85	3.45	0.46	16.20
Chronological Age (years)		11.61	2.75	7.71	16.91
Skeletal Age (years)		12.19	3.10	7.05	18.00
Relative Age (years)		−0.57	1.19	−3.37	3.01
Weight (kg)		44.63	17.56	19.70	109.70
Height (cm)		148.77	14.88	118.25	184.00
Body Mass Index Percentile		56.31	30.24	0.51	98.97
Waist Circumference (cm)		69.98	12.51	51.35	116.95
Waist to Height Ratio		0.47	0.06	0.36	0.68
Total % Body Fat (%)		26.62	7.54	13.27	42.60
SBP (mmHg)		99.31	8.98	80.0	128.0
DBP (mmHg)		59.19	9.86	40.0	84.0
Triglycerides (mg/dL)		92.56	65.80	32.0	580.0
Total cholesterol (mg/dL)		169.41	28.21	113.0	273.0
HDL-C (mg/dL)		53.82	10.35	28.0	79.0
LDL-C (mg/mL)		97.51	25.31	54.0	198.0
Fasting glucose (mg/dL)		83.49	6.25	71.2	103.2
Insulin (uIU/mL)		13.53	6.91	3.1	36.5
HOMA-IR		2.81	1.50	0.59	8.43
Duration (years) *		4.67	2.30	1.00	9.00
Categorical variable		N	%		
Relative Age	Earlier Maturation	14	15.91		
	Normal Maturation	61	69.32		
	Later Maturation	13	14.77		
Body Mass Index Percentile	Underweight	3	3.41		
	Normal or Healthy Weight	64	72.73		
	Overweight	10	11.36		
	Obese	11	12.50		

* Duration variable was calculated for participants with >1 observation among 52 participants with serial data.

HOMA-IR: Homeostatic model assessment for insulin resistance

**Table 2.** Longitudinal analysis – Mixed effect linear model analyses for log-transformed AMH

	Independent Variable ^a^	Beta Coefficient	SE	p-value	Model −2 Log Likelihood p-value
**Model 0**	Age	0.067	0.511	0.896	**256.3** *
	Age^2^	−0.018	0.041	0.665	**<0.0001**
	Age^3^	0.001	0.001	0.458	
**Model 1**	Relative Age	−0.089	0.030	**0.003**	**248.8**
	Age	0.202	0.526	0.702	**<0.0001**
	Age^2^	−0.031	0.042	0.468	
	Age^3^	0.001	0.001	0.292	
**Model 2**	Relative Age	−0.065	0.032	**0.042**	**241.9**
	Age	0.053	0.560	0.925	**<0.0001**
	Age^2^	−0.020	0.044	0.659	
	Age^3^	0.000	0.001	0.441	
	Total % Body Fat	0.008	0.006	0.178	
**Model 3**	Relative Age	−0.073	0.032	**0.023**	**247.3**
	Age	0.116	0.551	0.833	**<0.0001**
	Age^2^	−0.024	0.044	0.587	
	Age^3^	0.001	0.001	0.393	
	Total % Body Fat	0.008	0.006	0.162	
	Glucose	−0.008	0.004	**0.044**	
**Model 4**	Bone Age	0.901	0.385	**0.021**	**236.8** *
	Bone Age^2^	−0.085	0.031	**0.007**	**<0.0001**
	Bone Age^3^	0.002	0.001	**0.002**	

a Age effects were adjusted in the model as age, age-squared (Age^2^) and age-cubic variable (Age^3^)

* Likelihood ratio test (LRT) model 0 vs 1: 7.5, LRT model 4 vs model 1 = 12.0

## Data Availability

The datasets used and analyzed during the current study are available from the corresponding author on reasonable requests.
